# Development of a recombinase polymerase amplification combined with a lateral flow dipstick assay for rapid detection of the *Mycoplasma bovis*

**DOI:** 10.1186/s12917-018-1703-x

**Published:** 2018-12-20

**Authors:** Guimin Zhao, Peili Hou, Yanjun Huan, Chengqiang He, Hongmei Wang, Hongbin He

**Affiliations:** 1 Key Laboratory of Animal Resistant Biology of Shandong, Ruminant Disease Research Center, College of Life Science Shandong Normal University, No.88 Wenhua East Road, Lixia District, Jinan, 250014 Shandong Province China; 2 College of Animal Science and Technology, Qingdao Agricultural University, No.700 Changcheng Road, Chengyang District, Qingdao, 266109 Shandong Province China

**Keywords:** *Mycoplasma bovis*, Lateral flow dipstick, Recombinase polymerase amplification, Isothermal nucleic acid amplification, Rapid and visual detection

## Abstract

**Background:**

*Mycoplasma bovis* (*M. bovis*) is a major etiological agent of bovine mycoplasmosis around the world. Point-of-care testing in the field is lacking owing to the requirement for a simple, robust field applicable test that does not require professional laboratory equipment. The recombinase polymerase amplification (RPA) technique has become a promising isothermal DNA amplify assay for use in rapid and low-resource diagnostics.

**Results:**

Here, a method for specific detection of *M. bovis* DNA was established, which was RPA combined with lateral flow dipstick (LFD). First, the analytical specificity and sensitivity of the RPA primer and LF-probe sets were evaluated. The assay successfully detected *M. bovis* DNA in 30 min at 39 °C, with detection limit of 20 copies per reaction, which it was compared the real-time quantitative PCR (qPCR) assay. This method was specific because it did not detect a selection of other bacterial pathogens in cattle. Both qPCR and RPA-LFD assays were used to detect *M. bovis* 442 field samples from 42 different dairy farms in Shandong Province of China, also the established RPA-LFD assay obtained 99.00% sensitivity, 95.61% specificity, and 0.902 kappa coefficient compared with the qPCR.

**Conclusions:**

To the author’s knowledge, this is the first report using an RPA-FLD assay to visualise and detect *M. bovis*. Comparative analysis with qPCR indicates the potential of this assay for rapid diagnosis of bovine mycoplasmosis in resource limited settings.

**Electronic supplementary material:**

The online version of this article (10.1186/s12917-018-1703-x) contains supplementary material, which is available to authorized users.

## Background

*Mycoplasma bovis* (*M. bovis*) is a major etiological agent of bovine mycoplasmosis around the world. *M. bovis* has not only been confirmed as a major pathogen in bovine respiratory disease (BRD), but it has also causes disease in cattle of all ages, such as arthritis, otitis media, mastitis, and reproductive disorders [1]. Due to lack of effectiveness of treatments for controlling the disease in affected herds and decreasing growth rate of the cattle, it can result in serious economic losses in both dairy and beef cattle herds [2].

In 2008, a severe cattle respiratory disease was reported in Hubei Province of China, thereafter, it quickly spread to over 11 Chinese provinces. The organisms isolated from calf lungs were identified as *M. bovis* and designated strain Hubei-1. The 16S rRNA demonstrated 99.5% homology with *M. bovis* type strain PG45 [3]. Other places in China, such as Ningxia, Xinjiang, Guizhou, Chongqing, and Qingdao, later reported *M. bovis* isolated from dairy cows and beef cattle [4]. The lack of an effective control methods to prevent the rapid spread of *M. bovis* as well as its stubborn persistence on farms requires rapid and accurate diagnosis when clinical signs first appear [1].

The development of simple and quick nucleic acid detection methods could greatly improve diagnostics; however, practical on-site testing is infrequently done due to the lack of availability of robustly tested methods. Detection of *M. bovis* from clinical samples by traditional culture methods is quite time-consuming and is often hampered by bacterial contamination. Although detection technology of molecular biology for nucleic acids, such as polymerase chain reaction (PCR) and real-time quantitative PCR (qPCR) has shown high sensitivity and specificity, these methods need professional diagnostic laboratories and thermal cycling device. Such facilities may be lacking in disease epidemic and poor areas, especially in developing countries, the use of rapid on-site diagnostic methods would be extraordinary helpful in controlling bovine mycoplasmosis.

Recently, for DNA amplification, the recombinase polymerase amplification (RPA) technique has become a promising molecular technology for low-resource, rapid diagnostics [5]. Several types of tests have been described [6], and the RPA combined with lateral flow dipstick (LFD) appears especially suitable for point-of-care diagnosis in clinical specimens. The RPA assay has been reported for the rapid detection of Caprine arthritis-encephalitis virus, *Chlamydia trachomatis*, *Plasmodium falciparum* and other pathogens [7–9].

However, the detection of *M. bovis* using RPA-LFD assay has not yet been reported. In this study, we developed a rapid, sensitive, and on-site RPA combined with a LFD assay for the specific detection of *M. bovis* in the field.

## Results

### Evaluation of RPA nfo primer and probe sets

**Fig. 1:**
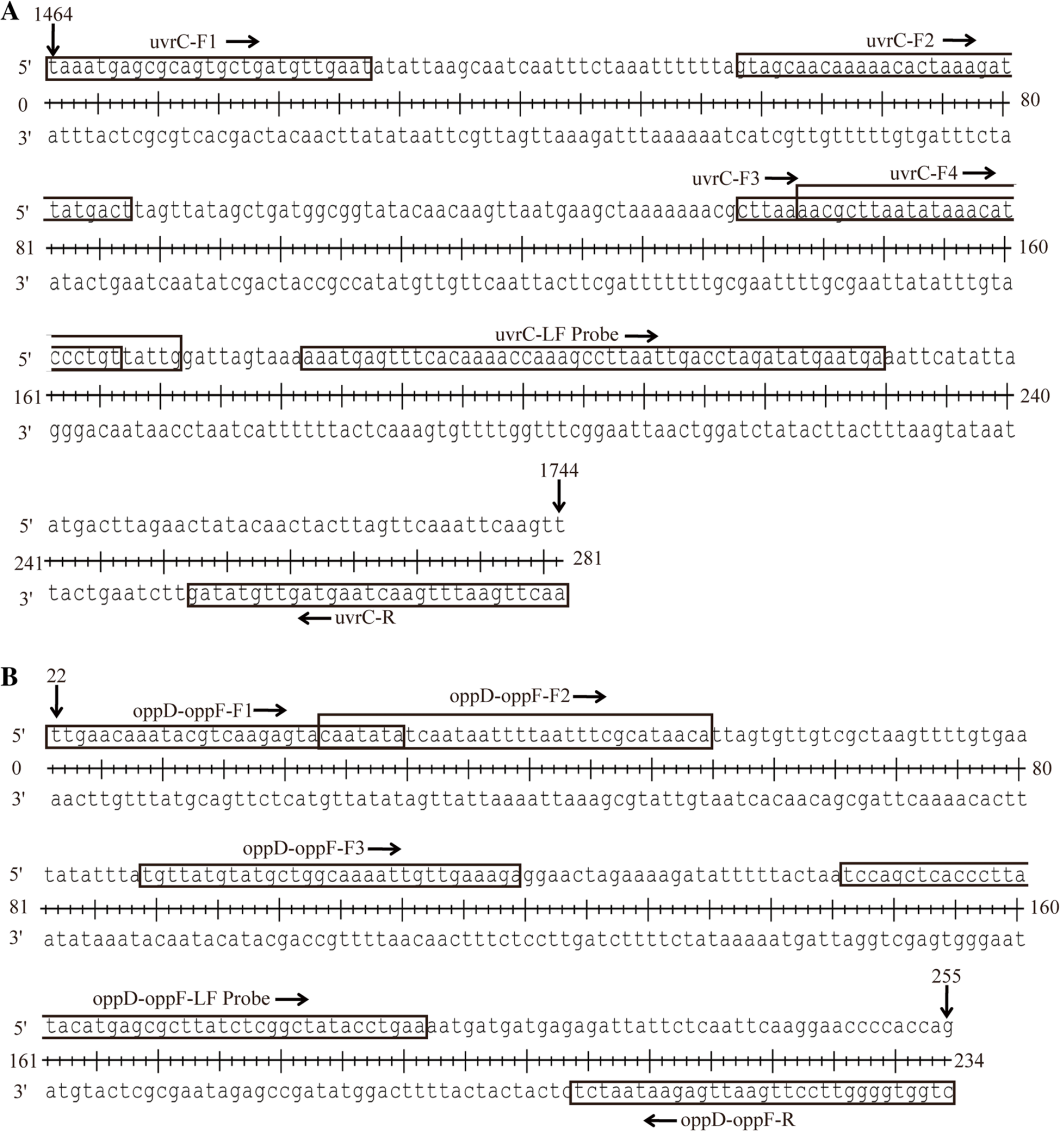
Details (location and sequence) for each primer and LF probe set used in our RPA-LFD assay. **a** The target region spanned nucleotides 1464–1744 of the *uvrC* gene of *Mycoplasma bovis* (GenBank Accession: AF003959.1); **b** The target region spanned nucleotides 22–255 of the *oppD-oppF* gene of *Mycoplasma bovis* (GenBank Accession: AF130119.1)

**Fig. 2:**
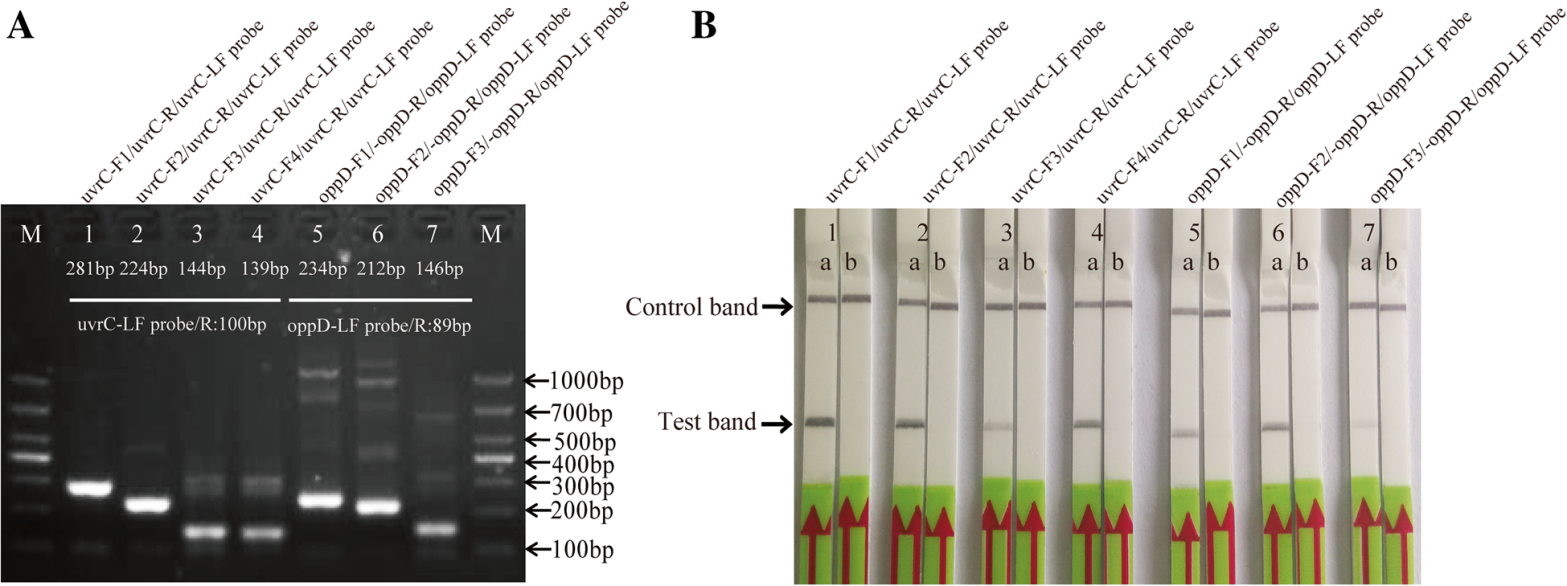
Screening of *Mycoplasma bovis* recombinase polymerase amplification combined with a lateral flow dipstick (RPA-LFD) primers and probe. **a**: The results of RPA-nfo reaction were detected by agarose-gel electrophoresis from seven set of primers and probe combination; **b**: ‘a’ showed results of RPA-nfo reaction by LFD test, and DNA template came from extracted *Mycoplasma bovis* reference type strain PG45 colonies, and ‘b’ was negative control (DNase-free water) that the corresponding combination of primers and probe. Samples were tested in triplicate with one reaction displayed in figure for each triplicate

The analytical specificity of seven primers combinations with LF-probes (Fig. 1 and Table 1) was confirmed using the genomic DNA extracted from *M. bovis* reference type strain PG45. The candidate primers for the RPA-LFD assay was screened with TwistAmp nfo reactions and preliminary analysis was performed on 2% agarose gel with labeled amplicons. The result showed that primer set uvrC-F1/uvrC-R/uvrC-LF probe yielded specific amplification efficiency for the RPA assay, and produced the expected size of the product of 281 base-pairs (Fig. 2a). The RPA-LFD test line appeared more quickly, within 5 min, and was more distinct than the other sets (Fig. 2b). This set (uvrC-F1/uvrC-R/uvrC-LF probe) was selected for subsequent evaluation (Fig. 1 and Table 1).

**Table 1:** Primer and probe sequences used for RPA-LFD assay

Name	Sequence (5′-3′)	Genome location	Amplification size (bp)
uvrC-F1	TAAATGAGCGCAGTGCTGATGTTGAAT	1464–1490	281
uvrC-F2	GTAGCAACAAAAACACTAAAGATTATGACT	1521–1550	224
uvrC-F3	CTTAAAACGCTTAATATAAACATCCCTGT	1601–1629	144
uvrC-F4	AACGCTTAATATAAACATCCCTGTTATTG	1606–1634	139
uvrC-R	Biotin-AACTTGAATTTGAACTAAGTAGTTGTATAG	1715–1744	
uvrC-LF Probe	FAM-AAATGAGTTTCACAAAACCAAAGCCTTAAT [dSpacer]GACCTAGATATGAATGA-C3 Spacers	1645–1692	100
oppD-oppF-F1	TTGAACAAATACGTCAAGAGTACAATATA	22–50	234
oppD-oppF-F2	CAATATATCAATAATTTTAATTTCGCATAACA	44–75	212
oppD-oppF-F3	TGTTATGTATGCTGGCAAAATTGTTGAAAGA	110–140	146
oppD-oppF-R	Biotin-CTGGTGGGGTTCCTTGAATTGAGAATAATCT	225–255	
oppD-oppF-LF Probe	FAM-TCCAGCTCACCCTTATACATGAGCGCTTATC [dSpacer]CGGCTATACCTGAA-C3 Spacers	167–212	89

*Abbreviations*: FAM is 6-Carboxyfluorescein, dSpacer is an exonuclease site, and C3 Spacers is a polymerase extension blocking site

### Determination of RPA reaction temperature and time

**Fig. 3:**
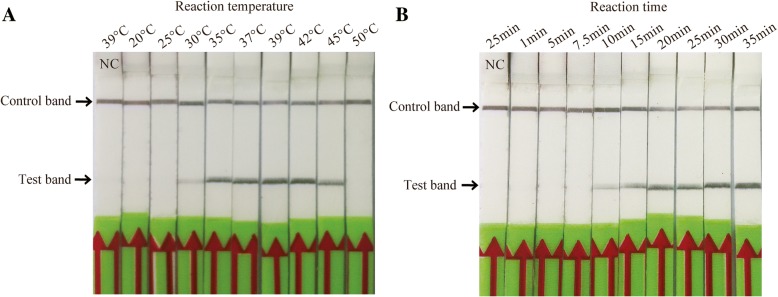
Optimization of incubation temperature and reaction time for *Mycoplasma bovis* recombinase polymerase amplification combined with a lateral flow dipstick (RPA-LFD) assay. **a** The amplification performance of RPA-LFD was effectively within the range of 35 °C to 45 °C. **b** Determination of amplification time. After 10 min of amplification reaction, the test line was clearly visible on the strip. Lane NC: negative control (DNase-free water). Samples were tested in triplicate with one reaction displayed in figure for each triplicate

The results showed that the RPA reaction could be determined at a wide range of temperatures from 30 to 45 °C. In addition, the test band was the brightest between 35 °C to 42 °C (Fig. 3a). Therefore, in the later the reaction of RPA-LFD assay temperature was set in 39 °C. Next, the optimum reaction time was estimated between 1 to 35 min. Results indicated that the most distinct band could be seen in the test zone position between 20 to 35 min with only weak bands seen after 10 min (Fig. 3b). According to the results, the incubation time was set at 30 min for the following RPA-LFD testing.

### Specificity and sensitivity of the *M. bovis* RPA-LFD assay

RPA specificity was tested using various Mycoplasma species from cattle and other pathogens that cause respiratory disease and mastitis (Table 2). RPA-LFD revealed high specificity and no cross-reaction was observed against other Mycoplasmas (*Mycoplasma agalactiae, Mycoplasma mycoides* subsp*. mycoides, Mycoplasma bovirhinis, Mycoplasma bovoculi, Mycoplasma bovigenitalium, Mycoplasma dispar, Mycoplasma canadense, Mycoplasma alkalescens, Mycoplasma canis, Mycoplasma arginini*), respiratory bacterial pathogens (*Pasteurella multocida, Mannheimia haemolytica, Trueperella pyogenes, Histophilus somni, Klebsiella pneumoniae*) and mastitis or other pathogens (*Staphylococcus aureus, Streptococcus agalactiae, Corynebacterium bovis, Pseudomonas aeruginosa, Proteus mirabilis, Enterobacter aerogenes, Brucella abortus, Escherichia coli*) (Additional file 1: Figure S1).

**Table 2:** *Mycoplasma bovis* and other bacterial species tested for specificity of the recombinase polymerase amplification combined with a lateral flow dipstick (RPA-LFD) assay

Number	Species	Strains/origin	RPA-LFD
1	*Mycoplasma bovis* PG45	ATCC25523^a^	Positive
2	*Mycoplasma bovis* Madison	ATCC27368^a^	Positive
3	*Mycoplasma bovis*	ATCC25025^a^	Positive
4	*Mycoplasma bovis* TJ14	Clinical separation^b^	Positive
5	*Mycoplasma bovis* SD16	Clinical separation^b^	Positive
6	*Mycoplasma agalactiae* PG2	BNCC132475^a^	Negative
7	*Mycoplasma mycoides* subsp. *mycoides*	BNCC126186^a^	Negative
8	*Mycoplasma bovirhinis* PG43	ATCC27748^b^	Negative
9	*Mycoplasma bovoculi*	ATCC29104^b^	Negative
10	*Mycoplasma bovigenitalium*	ATCC14173^b^	Negative
11	*Mycoplasma dispar*	ATCC27140^a^	Negative
12	*Mycoplasma canadense*	ATCC29418^a^	Negative
13	*Mycoplasma alkalescens*	ATCC29103^a^	Negative
14	*Mycoplasma canis* PG14	ATCC19525^a^	Negative
15	*Mycoplasma arginini*	ATCC23838^a^	Negative
16	*Pasteurella multocida* (A)	BNCC126487^a^	Negative
17	*Mannheimia haemolytica*	BNCC128674^a^	Negative
18	*Trueperella pyogenes*	Clinical separation^b^	Negative
19	*Histophilus somni*	Clinical separation^b^	Negative
20	*Klebsiella pneumoniae*	BNCC194477^b^	Negative
21	*Staphylococcus aureus*	Clinical separation^b^	Negative
22	*Streptococcus agalactiae*	BNCC185941^a^	Negative
23	*Brucella abortus* A19 vaccine strain	in our laboratory^b^	Negative
24	*Corynebacterium bovis*	BNCC131589^a^	Negative
25	*Escherichia coli* O157:H7	BNCC186579^a^	Negative
26	*Pseudomonas aeruginosa*	Clinical separation^b^	Negative
27	*Proteus mirabilis*	Clinical separation^b^	Negative
28	*Enterobacter aerogenes*	BNCC337113^a^	Negative

^a^These strains were purchased from BeNa Culture Collection biotechnology research institute (Beijing, China)

^b^These strains were preserved in our laboratory

**Fig. 4:**
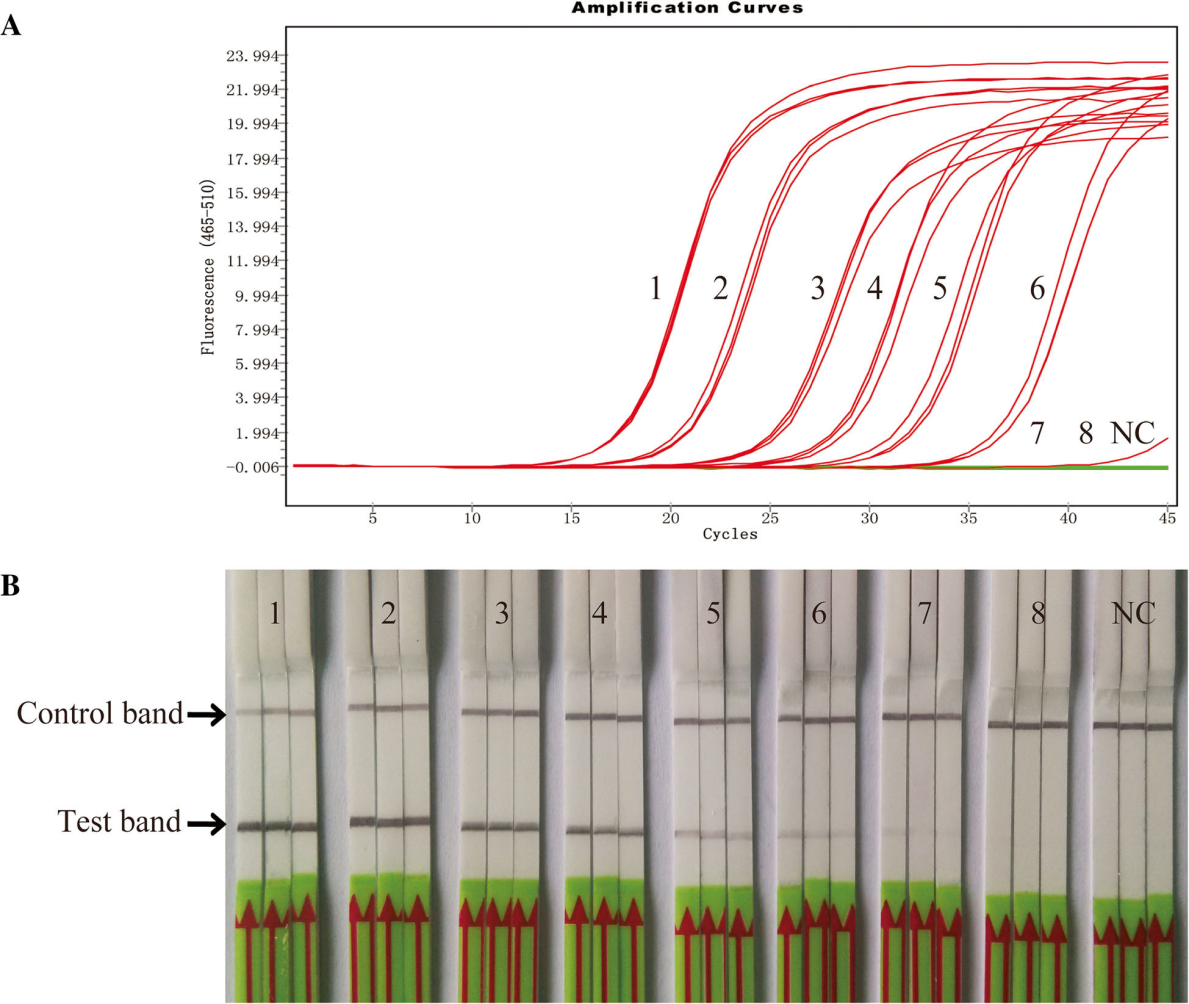
Comparison of sensitivities of the recombinase polymerase amplification combined with a lateral flow dipstick (RPA-LFD) and Real-time qPCR assays. Molecular sensitivity test results of the two assays were assessed using 10-fold serially diluted DNA as template. **a** Results by Real-time qPCR (with a detection limit of 83 copies/reaction DNA standards). **b** Results by RPA-LFD (with a detection limit of 20 copies/reaction DNA standards). Lane 1 to 8: 10-fold serially diluted *Mycoplasma bovis* plasmid DNA standards from 10^7^ to 10^0^ copies/uL. Lane NC: negative control (DNase-free water)

The sensitivity of RPA-LFD and qPCR assay was evaluated, and the repeatability test of limits of detection was showed in Table 3 and Fig. 4. Results from the testing of serially diluted plasmid DNA showed that RPA-LFD assay was capable of detecting 20 copies/reaction standards DNA, which was 4 times more sensitive than the qPCR assay (Fig. 4).

**Table 3:** Results of *Mycoplasma bovis* plasmid DNA standards detected by real-time qPCR and recombinase polymerase amplification combined with a lateral flow dipstick (RPA-LFD) assay

standards DNA (copies/ul)	Real-time qPCR			RPA-LFD		
	Ct 1	Ct 2	Ct 3	Test 1	Test 2	Test 3
NC	40.00	40.00	40.00	–	–	–
10^0^	40.00	40.00	40.00	–	+	–
10^1^	40.00	37.07	40.00	+	+	+
10^2^	36.76	36.79	36.41	+	+	+
10^3^	33.04	32.17	32.04	+	+	+
10^4^	28.63	28.02	28.30	+	+	+
10^5^	25.24	25.12	24.94	+	+	+
10^6^	20.30	20.76	20.71	+	+	+
10^7^	17.53	17.52	17.29	+	+	+

*NC* negative control (DNase-free water)

### Performance of RPA-LFD assay on clinical samples

RPA-LFD performance was analyzed using 442 clinical samples obtained from 42 different dairy farms in Shandong Province, China. The RPA-LFD and qPCR were executed in parallel. The RPA-LFD detected *M. bovis* DNA in 114 of 442 (25.79%) clinical samples while the qPCR assay found 100 (22.62%) of the same samples positive (Table 4). It indicated both assays were not significantly different using the independent-samples *t* test (*P* > 0.05). The established RPA-LFD assay yielded 99.00% sensitivity, 95.61% specificity, and 0.902 kappa coefficient with the qPCR (Table 5).

**Table 4:** Camparision of *Mycoplasma bovis* recombinase polymerase amplification combined with a lateral flow dipstick (RPA-LFD) assay and Real-time qPCR assay on clinical samples

Samples	Number of samples	RPA-LFD			Real-time qPCR		
		Positive	Negative	Positive rate (%)	Positive	Negative	Positive rate (%)
Nasal swabs	288	72	216	25.00	61	227	21.18
Fresh lungs	80	32	48	40.00	31	49	38.75
Joint fluids	32	4	28	12.50	4	28	12.50
Bulk tanks	42	6	36	14.29	4	38	9.52
Total	442	114	328	25.79	100	342	22.62

**Table 5:** Specificity, sensitivity, predictive value and kappa value of recombinase polymerase amplification combined with a lateral flow dipstick (RPA-LFD) and Real-time qPCR assays for diagnosing *Mycoplasma bovis* infection

		Real-time qPCR			
		Positive	Negative	Total	
RPA-LFD	Positive	99	15	114	92.11% (PPV)
	Negative	1	327	328	98.78% (NPV)
	Total	100	342	442	
		99.00%	95.61%	0.902	
		(Sensitivity)	(Specificity)	(Kappa coefficient)	

*PPV* Positive predictive value, *NPV* negative predictive value

## Discussion

Successful surveillance of bovine mycoplasmosis needs a rapid, specific and sensitive diagnostic method. The laboratory-based diagnosis techniques such as qPCR and enzyme linked immunosorbent assay (ELISA) are not able to meet the needs of a field test because the equipment required to perform them is not sufficiently portable or robust; furthermore some remote areas lack reliable power. With the development of isothermal nucleic acid amplification method, it is possible to perform on-site diagnostics in resource-limited settings. Here we established a new diagnostic assay based on RPA-LFD and evaluated its applicability to robustly and rapidly identify *M. bovis*, one of the crucial diseases affecting dairy and beef cattle herds in all over the world.

Rapid diagnostic tests for infectious diseases ordinarily use nucleic acid amplification technologies. Molecular tests for *M. bovis* have been developed based on the unique DNA sequences of the *uvrC* and *oppD/F* genes [10]. Subramaniam et al. (1998) [11] developed a PCR based on the DNA repair *uvrC* gene, which was shown to clearly differentiate between *M. bovis* and *M. agalactiae*. In this study, the analytical specificity of RPA primers and LF-probes based on *uvrC* and *oppD/F* gene was performed by agarose-gel electrophoresis. At the dSpacer position, when LF-probe is cleaved by nfo nuclease, the probe will be translated into a primer and act as priming for polymerase extension [6]. The one amplified fragment is from the different F primers and common R primers, and the other amplified fragment is LF-probe and common R primers. Therefore, the common R primers give two bands.

This paper describes a novel RPA-LFD assay based on LF-probe for *M. bovis* detection. Diagnostic specificity showed that the assay could detect reference type strain *M. bovis* PG45 and 4 others, but not other pathogens commonly found in cattle. Sensitivity revealed that the RPA-LFD assay was 4 times more sensitive than the qPCR method. To verify the diagnostic suitability of *M. bovis* RPA-LFD assay, the same clinical sample (*n* = 442) set was confirmed by the qPCR assay and yielded 0.902 kappa coefficient with the qPCR.

For diagnostic purposes, several isothermal molecular amplification technologies have been developed in the recent decade [12]. A comparison of 11 isothermal technologies indicates that RPA has some advantages over others. Firstly, RPA reaction is rapid, and nucleic acid amplification can be completed within 10–20 min. Secondly, it operates at lower temperatures (39 °C) than all comparable techniques with the exception of rolling cycle amplification (23 °C) [13]. Several published studies have performed RPA using a simple heat source system. Lillis et al. (2014) demonstrated that incubation of the RPA HIV-1 assay via ambient temperatures or using chemical heaters yields similar results to using electrically powered devices [14]. Moreover, the capacity of RPA to catalyze nucleic acid amplification using only body heat was also demonstrated [15]. A water bath is sufficient to carry out the RPA-LFD testing in the field or in less well-equipped laboratories [16]. Thirdly, commercial availability of freeze-dried reagents makes it easier operation in outside laboratory settings and in remote areas. Moreover, the RPA-LFD provides an easy to read visual signal for clinical point-of-care diagnosis. While PCR needs rapid and accurate temperature control during the amplification cycle, RPA can tolerates temperatures ranging from 35 to 42 °C without loss of reaction efficiency. This simplifies the instrument and reduces the cost [7]. One disadvantage however is the current price of testing: in China, this is estimated to be ¥75 CNY (about 11 USD) for the RPA-LFD and about ¥15 CNY (2 USD) for the qPCR reaction. The RPA-LFD is a new technology and it is believed that the cost will become cheaper as it becomes more widely used.

Further work is clearly necessary for this technology to be taken into the field including using body heat to activate the test and simplified DNA extraction possibly using NaOH DNA extraction [17].

## Conclusions

An RPA-LFD assay was developed for the rapid detection of *M. bovis* which will be suitable for on-farm testing and in particular in developing countries where sophisticated laboratory equipment is lacking. Its sensitivity and specificity were shown to be comparable to a previously published qPCR.

## Methods

### Strains and clinical samples

Reference type strain *M. bovis* PG45 was cultured as previously described [18], and originally purchased from BeNa culture collection biotechnology research institute (Beijing, China). A total of 442 clinical samples comprising nasal swabs (*n* = 288); fresh lungs (*n* = 80); joint fluids (*n* = 32); and bulk tank milk samples (*n* = 42) were collected between February 2015 and April 2017. Clinical samples from 400 cattle from 42 different dairy farms were examined, and the 42 farms were located in seventeen distinct geographic regions of Shandong province, China [19–21]. Bulk tank milk samples were used to estimate the apparent prevalence of *M. bovis* infection in 42 different dairy farms. A respiratory disease score was assigned and more than 6 points of cattle have at least two clinical symptoms of respiratory diseases, so they are considered sick [22]. The 288 nasal swab samples taken from all 42 dairy farms in which case suggests after BRD. The 80 fresh lung samples were sampled from postmortem cattle with BRD and the joint fluids were collected from 32 calves with arthritis. These clinical samples were tested for *M. bovis* by RPA-LFD and qPCR assay, in the ruminant disease research center laboratory

### DNA extraction

Approximately 2–4 colonies of the reference type strain *M. bovis* PG45 were transferred into 200 μl of sterile PBS using sterile disposable loop. The genomic DNA was extracted and eluted in 100 μl of sterile water by using bacterial genome DNA extraction kit (Tiangen Biotech Co., Ltd., Beijing, China) according to the manufacturer’s instructions. Then, DNA was maintained at − 20 °C until screening of the RPA-LFD primer and LF-probe could be performed. The genomic DNA of clinical samples (nasal swabs, fresh lungs and joint fluids) was extracted using TIANamp genomic DNA kit and TIANamp swab DNA kit (Tiangen Biotech Co., Ltd., Beijing, China), specific steps carried out in accordance with the instruction book. The genomic DNA of bulk tank milk samples from the pellet was extracted using TIANamp genomic DNA kit according to reported in literature [23].

### RPA primer and LF-probe design

The primers and LF-probe of the RPA reaction were used to amplify the *uvrC* gene (nucleotides 1464 to 1744 of the Genbank accession number: AF003959.1) and *oppD-oppF* gene (nucleotides 22 to 255 of the Genbank accession number: AF130119.1) sequences for *M. bovis*, and seven combinations of candidate primers (7 forward and 2 reverse) and two LF-probes were designed according to this two gene sequences. The details were shown in Fig. 1 and Table 1. The analytical specificity was performed using BLAST and no matches with other bacteria.

### RPA reaction and lateral flow dipstick (LFD) assay

Each 50 μl reaction volume was performed according to manufacturer’s instructions (TwistAmp nfo kit), and the volume of primer and LF-probe (10 μM) was adjusted accordingly. The following ingredients remained the same: 29.5 μl rehydration buffer, 2 μl DNA template, and 2.5 μl of 280 nM magnesium acetate. The test tubes were incubated at 39 °C for 25 min in a thermostatic water tank (Shanghai JingHong laboratory Co., Ltd., China). RPA amplification products were purified and the size of the RPA oligonucleotides was detected by 2% agarose-gel electrophoresis. Visualization of RPA amplicons was performed using LFD (HybriDetect, Milenia Biotec GmbH, Germany) according to manufacturer’s instructions.

### RPA conditions and optimization

To achieve optimal primers and LF-probe, different combinations were analysed for specificity and sensitivity detection. Next, the incubated temperature and time of RPA reaction were assessed according to the reagent instruction (TwistDX); temperature ranges were 20 °C to 50 °C, and time range was 1 min to 35 min.

### Specificity and sensitivity of the RPA-LFD assay

The diagnostic specificity of RPA-LFD assay was confirmed with DNA from various reference bacteria strains and strains from clinical cases (Table 2). Most of bacteria species were supplied by BNCC using their recommended media. The forward primer (uvrC-F1): 5’-TAAATGAGCGCAGTGCTGATGTTGAAT-3′ and the reverse primer (uvrC-R2): 5’-AACTTGAATTTGAACTAAGTAGTTGTATAG-3′ were used to amplify 281 bp of the *uvrC* gene of *M. bovis* (1464–1744 of Genbank accession number AF003959.1). The amplified fragment was ligated into plasmid pEASY-T3 cloning vector (Beijing TransGen Biotech Co., Ltd., Beijing, China). The DNA copy number was calculated as described in literature [24]. The analytical sensitivity of the RPA-LFD assay and qPCR assay was tested on standard plasmid DNA diluted in 10-fold serial steps from 10^7^ to 1 copies/ul. For the RPA-LFD assay, in order to evaluate the repeatability limits of detection, the standard plasmid DNA dilutions were tested in duplicates, and this was repeated three times.

### Real-time qPCR assay

The real-time qPCR assay for *M. bovis* was carried out to amplify a 170 bp sequences between 370 and 538 regions of *urvC* gene. The DNA molecular standard was prepared as previously described [5]. The qPCR amplification condition were as previously described [25, 26]. Briefly, the reaction was prepared as a 20 μl reaction volume containing 2 × Probe qPCR Mix, 0.8 μl of each 25 μM Mb-F: 5’-CAAAAGCAAAATGTTAAATTCAGG-3′ and Mb-R: 5’-CATATATAAGTGAGACTAACTTATT-3′, 0.8 μl of 7.5 μM probe: 5’-FAM-CAAAAGCAAAATGTTAAATTCAGG-BHQ2–3′ and 2 μl of DNA template. The thermal cycling parameters were as previously described [25, 26].

### Statistical analysis

Statistical analysis was performed using SPSS 16.0 software (Chicago, IL, USA); also independent-samples *t* test was used for evaluation of the results. For all analyses, *P* < 0.05 was considered significant. The diagnostic performance of the RPA-LFD and qPCR assays was assessed as described previously [27–29]. The kappa coefficient was defined as (Po − Pe)/(1 − Pe) [28].

## Additional file

Additional files 1: **Figure S1.** Specificity of the recombinase polymerase amplification combined with a lateral flow dipstick (RPA-LFD) assay. The specificity of the *Mycoplasma bovis* RPA-LFD assay was assessed for other bacterial pathogens genome cDNA of cattle that present similarly in the clinic. Lanes +: positive control (*Mycobacterium bovis* PG45), Lane NC: negative control (DNase-free water), Lanes 1: *Mycobacterium bovis* (Clinical separation), Lanes 2 to 23: *Mycoplasma agalactiae*, *Mycoplasma mycoides* subsp. *mycoides*, *Mycoplasma bovirhinis*, *Mycoplasma bovoculi*, *Mycoplasma bovigenitalium*, *Mycoplasma dispar*, *Mycoplasma canadense*, *Mycoplasma alkalescens*, *Mycoplasma canis*, *Mycoplasma arginini*, *Pasteurella multocida*, *Mannheimia haemolytica*, *Trueperella pyogenes*, *Histophilus somni*, *Klebsiella pneumoniae*, *Staphylococcus aureus*, *Streptococcus agalactiae*, *Corynebacterium bovis, Pseudomonas aeruginosa*, *Proteus mirabilis*, *Enterobacter aerogenes*, *Brucella abortus,* and *Escherichia coli,* respectively. Samples were tested in triplicate with one reaction displayed in figure for each triplicate. (DOCX 1581 kb)

## Abbreviations

BRD: Bovine respiratory disease
ELISA: Enzyme linked immunosorbent assay
LFD: Lateral flow dipstick
PCR: Polymerase chain reaction
qPCR: Real-time quantitative PCR
RPA: Recombinase polymerase amplification
