# Supplementary material for: Development of a recombinase polymerase amplification combined with a lateral flow dipstick assay for rapid detection of the Mycoplasma bovis
Source: BMC Vet Res. 2018 Dec 20;14:412. doi: 10.1186/s12917-018-1703-x (PMC6302395; doi:10.1186/s12917-018-1703-x)
Supplement: Supplementary file 1 — Figure S1. Specificity of the recombinase polymerase amplification combined with a lateral flow dipstick (RPA-LFD) assay. The specificity of the Mycoplasma bovis RPA-LFD assay was assessed for other bacterial pathogens genome cDNA of cattle that present similarly in the clinic. Lanes +: positive control (Mycobacterium bovis PG45), Lane NC: negative control (DNase-free water), Lanes 1: Mycobacterium bovis (Clinical separation), Lanes 2 to 23: Mycoplasma agalactiae, Mycoplasma mycoides subsp. mycoides, Mycoplasma bovirhinis, Mycoplasma bovoculi, Mycoplasma bovigenitalium, Mycoplasma dispar, Mycoplasma canadense, Mycoplasma alkalescens, Mycoplasma canis, Mycoplasma arginini, Pasteurella multocida, Mannheimia haemolytica, Trueperella pyogenes, Histophilus somni, Klebsiella pneumoniae, Staphylococcus aureus, Streptococcus agalactiae, Corynebacterium bovis, Pseudomonas aeruginosa, Proteus mirabilis, Enterobacter aerogenes, Brucella abortus, and Escherichia coli, respectively. Samples were tested in triplicate with one reaction displayed in figure for each triplicate. (DOCX 1581 kb) [file 12917_2018_1703_MOESM1_ESM.docx]

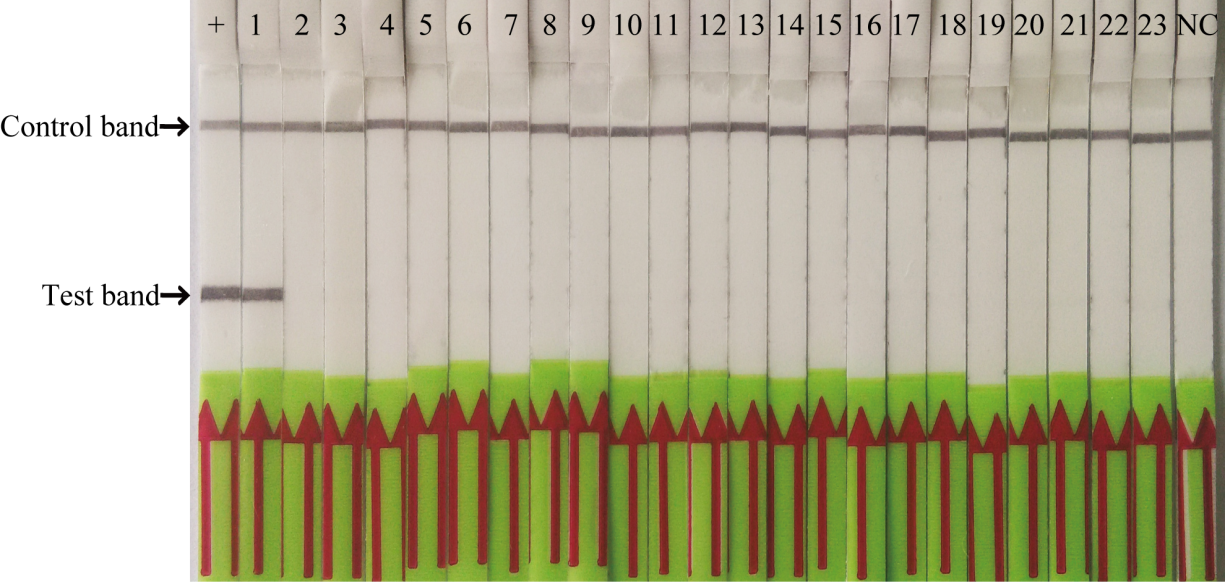


**Figure S1.** Specificity of the RPA-LFD assay. The specificity of the *Mycoplasma bovis* RPA-LFD assay was assessed for other bacterial pathogens genome cDNA of cattle that present similarly in the clinic. Lanes +: positive control (*Mycobacterium bovis* PG45), Lane NC: negative control (DNase-free water), Lanes 1: *Mycobacterium bovis* (Clinical separation), Lanes 2 to 23: *Mycoplasma agalactiae, Mycoplasma mycoides* subsp*. mycoides, Mycoplasma bovirhinis, Mycoplasma bovoculi, Mycoplasma bovigenitalium, Mycoplasma dispar, Mycoplasma canadense, Mycoplasma alkalescens, Mycoplasma canis, Mycoplasma arginini*, *Pasturella multocida, Mannheimia hemolytica, Trueperella pyogenes, Histophilus somni, Klebsiella pneumoniae, Staphylococcus aureus, Streptococcus agalactiae, Corynebacterium bovis, Pseudomonas aeruginosa, Proteus mirabilis, Enterobacter aerogenes, Brucella abortus,* and *Escherichia coli*, respectively. Samples were tested in triplicate with one reaction displayed in figure for each triplicate.
